# Targeting Cyclin-Dependent Kinases for Treatment of Gynecologic Cancers

**DOI:** 10.3389/fonc.2018.00303

**Published:** 2018-08-08

**Authors:** Z. Ping Lin, Yong-Lian Zhu, Elena S. Ratner

**Affiliations:** Department of Obstetrics, Gynecology, and Reproductive Sciences, Yale University School of Medicine, New Haven, CT, United States

**Keywords:** gynecologic cancer, homologous recombination, cell cycle, cyclin-dependent kinase, ribonucleotide reductase, cdc25 phosphatase, small molecule inhibitor

## Abstract

Ovarian, uterine/endometrial, and cervical cancers are major gynecologic malignancies estimated to cause nearly 30,000 deaths in 2018 in US. Defective cell cycle regulation is the hallmark of cancers underpinning the development and progression of the disease. Normal cell cycle is driven by the coordinated and sequential rise and fall of cyclin-dependent kinases (CDK) activity. The transition of cell cycle phases is governed by the respective checkpoints that prevent the entry into the next phase until cellular or genetic defects are repaired. Checkpoint activation is achieved by p53- and ATM/ATR-mediated inactivation of CDKs in response to DNA damage. Therefore, an aberrant increase in CDK activity and/or defects in checkpoint activation lead to unrestricted cell cycle phase transition and uncontrolled proliferation that give rise to cancers and perpetuate malignant progression. Given that CDK activity is also required for homologous recombination (HR) repair, pharmacological inhibition of CDKs can be exploited as a synthetic lethal approach to augment the therapeutic efficacy of PARP inhibitors and other DNA damaging modalities for the treatment of gynecologic cancers. Here, we overview the basic of cell cycle and discuss the mechanistic studies that establish the intimate link between CDKs and HR repair. In addition, we present the perspective of preclinical and clinical development in small molecule inhibitors of CDKs and CDK-associated protein targets, as well as their potential use in combination with hormonal therapy, PARP inhibitors, chemotherapy, and radiation to improve treatment outcomes.

## Introduction

Ovarian, uterine/endometrial, and cervical cancers are three major malignancies of reproductive organs in women in US. It is estimated that 76,470 new cases of uterine and cervical cancers and 22,240 new cases of ovarian cancer would be diagnosed in 2018. However, ovarian cancer is the most lethal gynecologic cancer accountable for estimated 14,070 deaths surpassing 11,350 deaths for uterine cancer and 4,170 deaths for cervical cancer, according to Cancer Statistics (1). At the global level, ovarian cancer contributed to 161,100 deaths in 2015 (2). Cervical cancer is mostly caused by human papillomavirus (HPV) through sexual transmission whereas the causes of uterine and ovarian are much perplex and await further investigation. Recent advance in targeted therapies and rational combination modalities have proven to deliver improved treatment outcomes for gynecologic cancers over traditional chemotherapy. The new therapeutic approaches are made possible through a better understanding of molecular/genetic basis of cancer biology and advent of innovative technologies to target the vulnerability of cancers. De-regulation of cell cycle is the hallmark of cancer development and malignant progression. In this review, we will discuss preclinical development and clinical trials of small molecule inhibitor drugs targeting defects in cell cycle regulation and its associated DNA repair pathways for treatment of gynecologic cancers.

## Cell cycle and regulation-the basic

Cell cycle is a process of successive distinct events that lead to accurate duplication of genetic materials and equal division of a cell [for review, see (3)]. In eukaryotic cells, cell cycle comprises four major periods defined as G1, S, G2, and M phases. The G1 is the first growth phase of the cell cycle during which cells undergo active synthesis of proteins and cellular components, as well as increasing the number of organelles and the size of cells. The S phase is the synthesis phase of the cell cycle when DNA synthesis takes place and results in duplication of all chromosomes. The G2 phase is the second growth phase of the cell cycle. During this period, cells continue protein synthesis in preparation for mitosis. The M phase is the final phase of the cell cycle during which cell undergo cell division to separate duplicated chromosomes and cellular components, resulting in two identical daughter cells.

**Figure 1:**
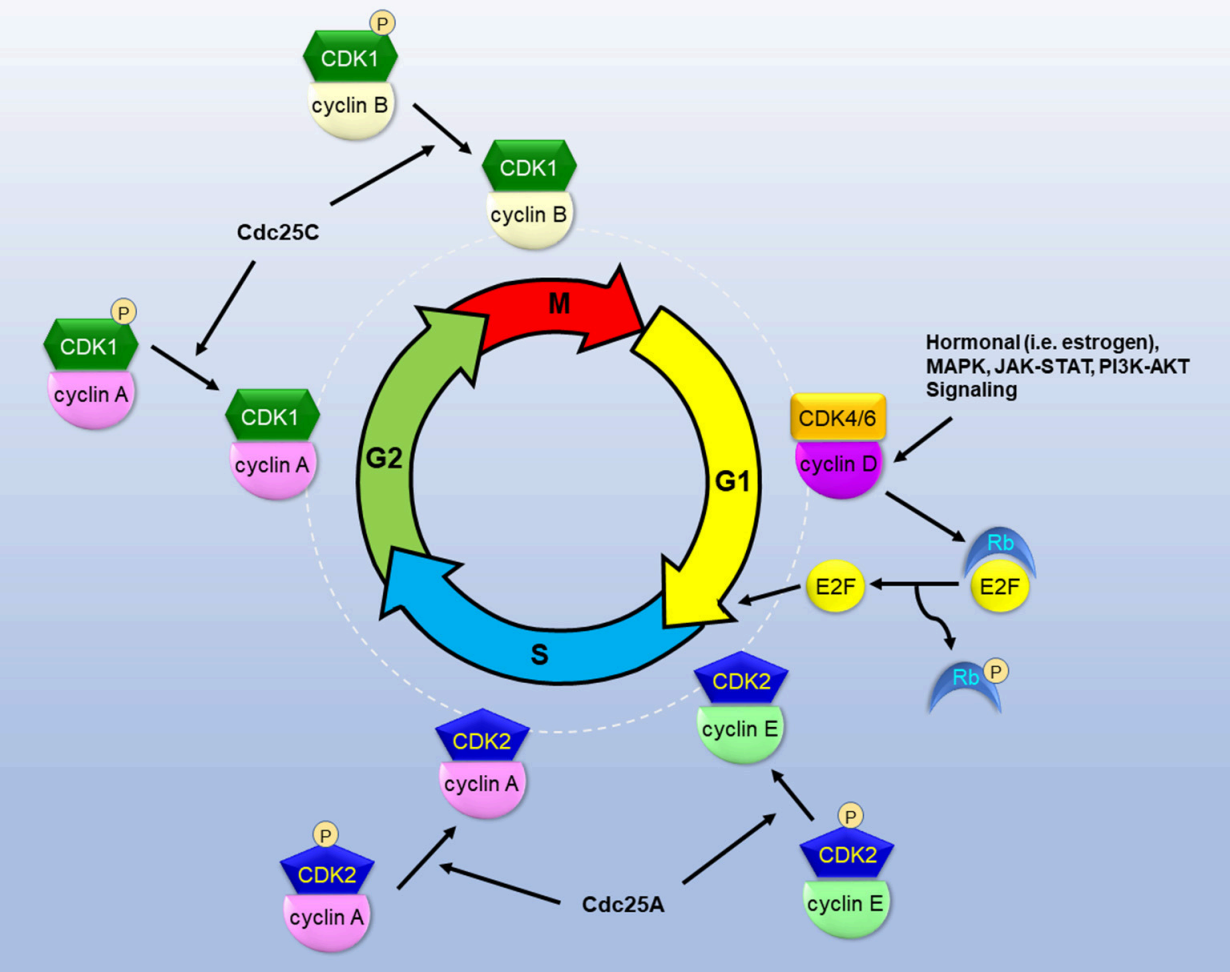
The roles of CDKs in the cascade of cell cycle. CDKs bind to specialized cyclins to form active complexes that drive cell cycle phase progression and transition into next phases. Growth and mitogenic signals induce cyclin D and activate CDK4, thereby inactivating Rb and releasing E2F to instigate G1 phase progression. Cdc25 phosphatases dephosphorylate and activate CDKs to promote S/G2/M phase progression.

The progression of cell cycle is regulated by sequential and coordinated rise and fall of cyclin-dependent kinase (CDK) activity (Figure 1). Regulatory cyclins bind to catalytic CDKs to form activated complexes. During the G1 phase, cyclin D rises in response to growth/mitogenic stimuli and forms the complex with CDK4/6. The CDK4/6-cyclin D complex phosphorylates retinoblastoma susceptible protein (Rb), thereby causing the dissociation of the transcription factor E2F from Rb (4). Thus, activated E2F commits cells to the S phase entry by mediating the transcription of cyclins E and A, and other proteins, including ribonucleotide reductase (RNR), thymidylate synthase, dihydrofolate reductase, and DNA polymerases, necessary for DNA synthesis. Cyclin E binds to CDK2 to form the CDK2-cyclin E complex which commences the S phase progression. To promote S phase progression, Cdc25A phosphatase activates CDK2-cyclin E by removing inhibitory phosphates from CDK2. Cyclin A later replaces cyclin E to form the CDK2-cyclin A complex which is required for passage through the S phase. During the G2 phase, CDK1 displaces CDK2 to form the CDK1-cyclin A complex. Cdc25C is responsible for dephosphorylating and activating CDK1-cyclin A to facilitate G2 phase progression. During the M phase, cyclin B binds to CDK1 to form the CDK1-cyclin B complex which promotes mitosis. The spindle assembly checkpoint (SAC) functions to inactivate the anaphase-promoting complex (APC/C) and prevent mitosis from metaphase to anaphase. PLK1 and Aurora B kinases are involved in the regulation of mitotic progression through coordination with the SAC (5, 6).

The transition between cell cycle phases is governed by G1, G2, and M (mitotic) phase checkpoints to prevent the entry into the next phase until the defects are amended [for review, see (7)]. The G1 phase checkpoint is controlled by CDK4/6-cyclin D-mediated phosphorylation of Rb and subsequent transcriptional activation of E2F. The activation of the G1 checkpoint occurs to prevent the transition to the S phase in response to inadequate or inhibitory growth signals, hypoxia, and DNA damage. Ataxia telangiectasia mutated (ATM) or ataxia telangiectasia and Rad3 related (ATR) function as sensors for DNA damage and phosphorylate Chk2 and Chk1, respectively. Phosphorylated Chk1 and Chk2 in turn target phosphorylation of Cdc25A for degradation and inactivates CDK2-cyclin E. Therefore, the cell cycle is transiently arrested in the G1 phase. To maintain the arrest, Chk1 and Chk2 phosphorylate and stabilize the tumor suppressor p53 that in turn leads to transcription of p21 (CDKN1A), a CDK2-cyclin E inhibitor, or CDKI. The G1 checkpoint also involves several CDKIs including p16 and p27. p16 (CDKN2A) acts to inhibit CDK4/6-cyclin D and E2F transcriptional activity, thereby blocking G1 to S phase transition. p27 (CDKN1B) binds to and inactivates CDK2-cyclin E or CDK4-cyclin D, thereby halting cell cycle at the G1 phase. The G2 phase checkpoint serves as a safeguard to prevent the entry into M phase in response to incomplete DNA replication and DNA damage. DNA damage results in phosphorylation and activation of Chk1, which in turn phosphorylates and stabilizes Wee1. Wee1 kinase causes inhibitory phosphorylation of CDK1 at its Thr14 and Tyr15, thereby inactivating CDK1-cyclin B. Concurrently, Chk1 phosphorylates Cdc25C and inhibits its phosphatase activity for activating CDK1-cyclin B. p53 also acts to augment the G2 checkpoint by inducing 14-3-3 which binds to and exports phosphorylated CDK1-cyclin B from the nucleus. As a result, this multifaceted abrogation of CDK1-cyclin B activity blocks mitotic entry and arrests cells at the G2 phase until DNA damage is repaired. The M phase checkpoint prevents segregation of duplicated chromosomes that are not properly aligned and anchored to spindle microtubules at the metaphase plate (8). CDK1-cyclin B phosphorylates and activates APC/C to promote mitosis. However, improper attachment of kinetocores to spindle microtubules activates the SAC by inhibiting the APC/C-Cdc20 complex and thus preventing the progression from metaphase to anaphase until chromosomes are properly attached.

## DNA repair during cell cycle phases

With the purpose of maintaining genomic stability, cell cycle regulation is controlled by checkpoints largely in response to DNA damage and replication stress. DNA double strand breaks (DSBs) represent the most severe and lethal type of DNA damage. Cells have evolved an array of sophisticate DNA damage repair machinery that counteract the deleterious effects of DSBs occurring at various cell cycle phases in a timely and coordinated, if not competitive, manner.

**Figure 2:**
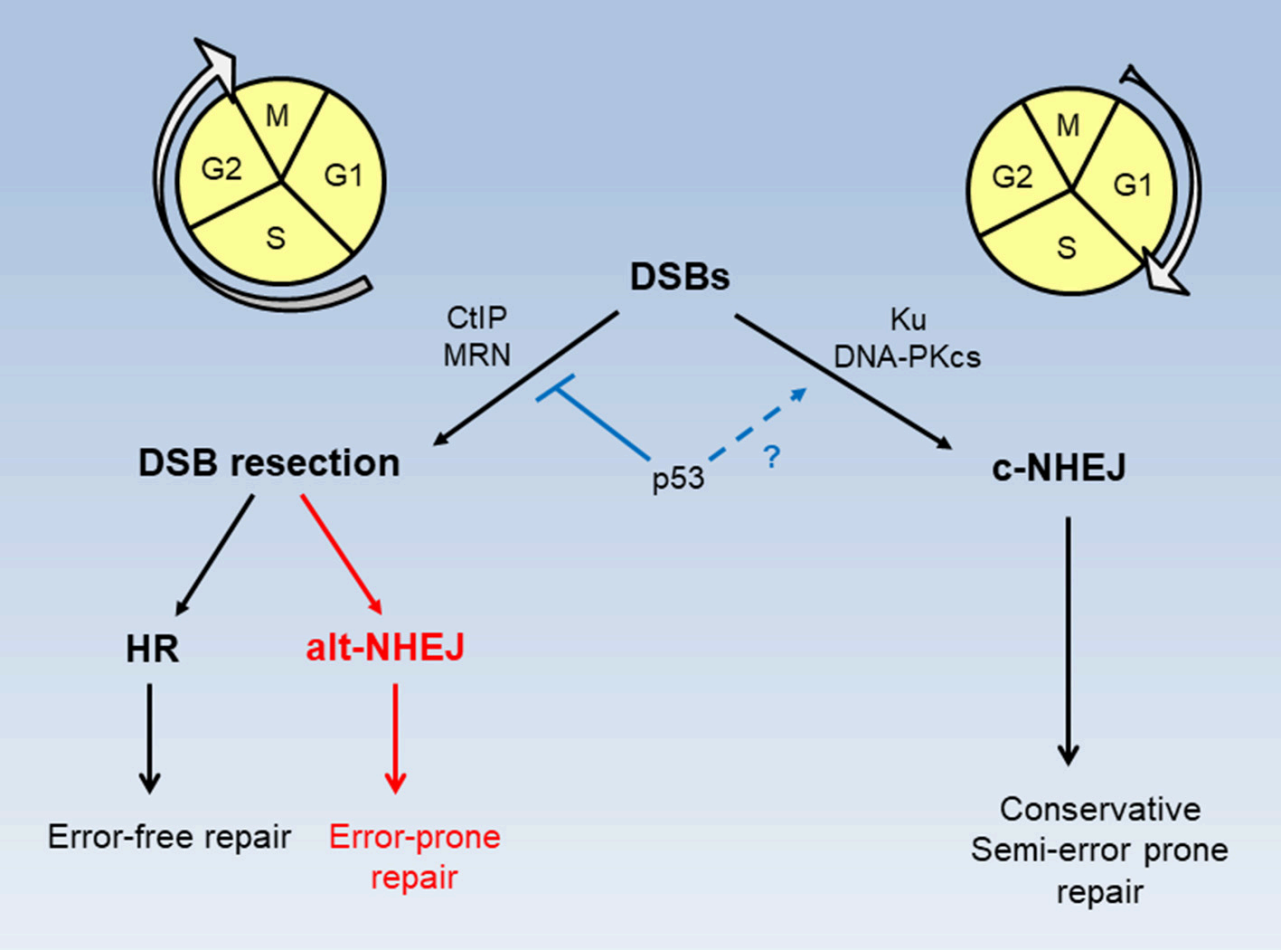
Modes of DSB repair in cell cycle phases. DSB repair by HR and alt-NHEJ requires CtIP and MRN activity and takes place in S and G2 phases of the cell cycle. DSB repair by c-NHEJ occurs predominately, but not exclusively, in the G1 phase. p53 functions to restrict DSB resection for HR and alt-NHEJ, while possibly promoting accurate c-NHEJ.

Homologous recombination (HR) and non-homologous end joining (NHEJ) are two major pathways for the repair of DSBs (Figure 2). HR operates strictly in S and G2 phases of the cell cycle only when homologous sister chromatids are present, while NHEJ which functions independently of homologous chromosomal sequences can occur throughout the cell cycle (9). HR is an error-free DSB repair process while NHEJ is generally considered error-prone. NHEJ can be further divided into two sub-pathways, canonical NHEJ (c-NHEJ) occurring in G1 phase and alternative NHEJ (alt-NHEJ) occurring in S and G2 phases (10). In fact, c-NHEJ is a conservative repair necessary for physiological processes including class switch recombination and V(D)J recombination (11, 12). Recent studies suggest that the fallibility of collective NHEJ is mainly attributed to alt-NHEJ, a highly error-prone and mutagenic repair that causes large deletions and chromosomal rearrangement (13). Interestingly alt-NHEJ shares the common characteristics with HR in the requirement of DSB resection. With versatile nature and swift process, c-NHEJ predominates in DSB repair to quickly and effectively restore and maintain genomic integrity in mammalian cells (11, 14–17). The binding of Ku factors, key components of c-NHEJ, to DSB ends by default limits extensive resection to prevent HR and alt-NHEJ (18, 19). Conversely extensive resection of DSB ends prevents Ku factors from binding and allows HR and alt-NHEJ to occur (10). Wild type p53 can inhibit error-prone alt-NHEJ to ensure accurate re-ligation of DSBs by c-NHEJ (20, 21). A large body of evidence indicates that a loss of p53 function leads to increased HR activity in cancer cells (22–29). A study of siRNA screening demonstrates that silencing of BRCA1, BRCA2, Rad51, and HR-associated genes selectively sensitizes p53-deficient cancer cells to cisplatin (30) that incurs DSBs secondary to primary DNA adducts (31–33).

## The link between cell cycle checkpoint and HR repair

Cell cycle checkpoints prevent cells from progressing to the next phase until DNA damage is repaired. However, the exact connection between checkpoint activation and DNA repair remains elusive. Cumulative evidence indicates that CDK activity is required for HR to mediate DSB repair during the S and G2 phases (34–37). It seems counterintuitive since inactivation of CDK is a critical step for checkpoint activation in response to DNA damage. To address this controversy, Buisson et al. has recently elucidated that initial high CDK activity serves to promote DSB end resection, which in turn activates ATR and Chk1 to inhibit CDK and concurrently stimulate the PALB2-mediated step in HR repair (38). CDKs phosphorylate a plethora of protein substrates, such as CtIP and BRCA1, required for S and G2 progression. CtIP is a nuclear protein that interacts with Nbs1 of the MRN (Mre11-Rad50-Nbs1) complex to stimulate the nuclease activity of Mre11 for DSB end resection (39–41). The BRCT domain of BRCA1 binds to CtIP through CDK-mediated phosphorylation of CtIP at Ser327 (42–44), which has recently been shown to accelerates DSB end resection (45) and antagonize 53BP1-mediated NHEJ (46, 47). CDK also phosphorylates CtIP at Thr847 and activates CtIP for DSB end resection (35). Furthermore, ATR phosphorylates CtIP at Thr859 thereby rendering the binding of CtIP to chromatin for execution of DSB end resection (48). Extensive DSB end resection by MRN-CtIP leads to 3′-single strand DNA (ssDNA) overhands which are coated with RPA. The ssDNA-RPA complex recruits and activates ATR, thereby resulting in Chk1 activation (49). These lines of evidence suggest that CDK activity is critical for initiation and promotion of DSB end resection for checkpoint activation and HR repair in a temporal and coordinated manner.

## Cell cycle de-regulation and gynecologic cancers

Normal cells depend on an array of cell cycle machinery to maintain timely and orderly progression of cell cycle phases. These sophisticate regulatory mechanisms of cell cycle is primed for action because replication errors and spontaneous DNA damage are constant threats to the integrity and survival of cells. By contrast, virtually most cancer cells display defects in cell cycle regulation and DNA repair which promote advantageous mutations, oncogenic growth, and uncontrolled cell proliferation.

Development of ovarian cancer involves several factors including genetics, hormones, and environment. Mutations in the p53 gene are the most frequent genetic events that occur in advanced ovarian cancer (50–53). More than 95% of high-grade serous ovarian cancer harbors mutated p53 (53). p53 deficiency abolishes the G1 checkpoint and leads to uncontrolled cell proliferation. In addition, loss of p53 function is permissive for BRCA mutation and HR repair deficiency that promote carcinogenesis, malignant progression, invasiveness in advanced breast and ovarian cancers (54). Mutations of Rb and amplification of cyclin D have also been found in ovarian cancer (55, 56). In cervical cancer, 93% cases are caused by HPV infection (57). The oncogenic properties of HPV are attributable to its E6 and E7 proteins (58). E6 forms the complex with p53 and target p53 for degradation. E7 binds to Rb and causes the release of E2F. Both viral oncogenic proteins effectively abrogate the G1 checkpoint, thereby resulting in cell transformation and proliferation. Genetic defects and hormonal imbalance are also considered the primary causes of uterine/endometrial cancer. With regard to cell cycle de-regulation, mutations in p53, loss of p16 expression, and amplification of cyclin D are among genetic alterations that underlie the development and progression of uterine/endometrial cancers (59–61).

Discovery and development of small molecular inhibitors targeting ablation of CDK activity has long been an interest in academic research and pharmaceutical industry. This pharmacological approach will restrict hormone-dependent CDK-driven phase transition and curb uncontrolled cell proliferation in cancers. Furthermore, CDK-mediated DNA repair process can be exploited as a synthetic lethal target to enhance the efficacy of DNA damaging modalities including PARP inhibition therapy. In the following topics, we will discuss therapeutic strategies of blocking CDK activity by direct and indirect pharmacological inhibition with three classes of small molecule compounds (Table 1). Preclinical and clinical development of these small molecule inhibitors mainly within the scope of combination therapies will also be discussed.

**Table 1:** Examples of preclinical and ongoing clinical development of small molecule Inhibitors that target CDKs, Cdc25, and RNR.

**Inhibitors**	**Target**	**Phase of development**	**Drug in combination**	**Disease/cancer cell type**	**References/ClinicalTrials.gov Identifier**
Ribociclib	CDK4/6	FDA-approved	Letrozole	Breast cancer	(65)
		Phase II	Letrozole and temsirolimus	Endometrial cancer	NCT03008408
		Phase II	Letrozole	Ovarian and endometrial cancers	NCT02657928
		Phase I	Carboplatin and paclitaxel	Ovarian cancer	NCT03056833
Palbociclib	CDK4/6	FDA-approved	Letrozole	Breast cancer	(66)
Abemaciclib	CDK4/6	FDA-approved	Fulvestrant	Breast cancer	(68)
Roscovitine	CDK1/2	Phase I	Sapacitabine	Pancreatic, breast, ovarian cancers	NCT00999401
Dinaciclib	CDK1/2	Phase I	Veliparib	Breast cancer	NCT01434316
Ro-3306	CDK1/2	Preclinical	Olaparib	Breast cancer	(75)
		Preclinical	Rucaparib	Non-small cell lung cancers	(74)
BMS-387032	CDK1/2	Preclinical	Cytarabine	Acute myeloid leukemia	(87)
PHA-793887	CDK1/2	Preclinical	Radiation	Cervical cancer	(38, 88)
AZD5438	CDK1/2	Preclinical	Radiation	Non-small cell lung cancer	(89, 90)
LGH00031	Cdc25	Preclinical	–	Various cancers	(96)
BN82002	Cdc25	Preclinical	–	Various cancers	(97)
LB100	Cdc25	Preclinical	Radiation, daunorubicin, cisplatin	Ovarian and various cancers	(98–102)
Hydroxyurea	RNR	FDA-approved	Radiation	Head and neck cancers	(109)
Triapine	RNR	Phase II	Cisplatin and radiation	Cervical and vaginal cancers	NCT02466971
		Preclinical	Platinum, doxorubicin	Ovarian Cancer	(28, 108)
		Preclinical	Olaparib, etoposide	Ovarian Cancer	(107)

## CDK inhibitors

Targeted inhibition of CDKs recapitulates the effects of checkpoint activation to counteract aberrant and unrestricted progression of cell cycle phases in many cancers. Currently developed and clinically-approved CDK inhibitors can be divided into two categories based on their targets in cell cycle phases: G1-targeted CDK4/6 inhibitors and S/G2/M-targeted CDK1/2 inhibitors. Most of these small molecule compounds are ATP-competitive inhibitors and therefore some possess an overlapping spectrum of activity across subtypes of CDKs. CDKs are not directly involved in cell cycle but transcriptional regulation including CDK7, 8, 9, 11, 12, and 13.

Small molecule inhibitors of CDK4/6 have been demonstrated clinically effective in combination with hormonal/endocrine therapy against several types of cancers, especially breast cancer. Several oncogenic signaling pathways including steroid hormones, PI3K-AKT, JAK-STATs, and MAPKs are known to promote cell proliferation by inducing cyclin D1 and promoting CDK4/6 activity (62, 63). Given that the majority of breast cancer is initially positive for steroid hormone receptors (64), blockade of CDK4/6 activity by small molecule inhibitors represents a rational strategies and has proven efficacious in the treatment of breast cancer. For this reason, currently three orally active CDK4/6 inhibitors have been approved by FDA based on promising results from well-conducted clinical trials. Clinical studies of breast cancer leading to FDA approval demonstrated that CDK4/6 inhibitors in combination with endocrine therapy exhibited superior activity compared with endocrine therapy alone. Ribociclib (LEE011) is a selective CDK4/6 inhibitor approved for use in combination with the aromatase inhibitor letrozole to treat hormone receptor-positive advanced or metastatic breast cancer (65). Palbociclib (PD-0332991) is a selective CDK4/6 inhibitor approved for use in combination with letrozole to treat hormone receptor-positive advanced breast cancer as initial endocrine therapy (66). Palbociclib gained additional FDA approval for use in combination with the estrogen receptor degrader fulvestrant to treat hormone receptor-positive advanced or metastatic breast cancer in patients with disease progression after endocrine therapy (67). Abemaciclib (LY2835219) is a selective CDK4/6 inhibitor approved for use in combination with fulvestrant to treat hormone receptor-positive advanced or metastatic breast cancer in patients with disease progression following endocrine therapy (68). In addition to combination therapy, abemaciclib was approved by FDA as monotherapy for hormone receptor-positive advanced or metastatic breast cancer in patients with disease progression after endocrine therapy (69). These CDK4/6 inhibitors are generally well tolerated in both combination and monotherapy. The most common side effect of these inhibitors is neutropenia. Other hematological and GI side effects include anemia, nausea, diarrhea, anorexia, and fatigue. Thus, it is recommended that ribociclib and palbociclib should be used once daily in a 3-week on and 1-week off schedule, whereas abemaciclib can be used twice daily in a continuous manner (70).

The success of these FDA-approved CDK4/6 inhibitors for hormone receptor-positive breast cancer can serve as a paradigm for ongoing clinical development of therapy for gynecologic cancers because of some shared characteristics especially in hormonal dependency. Clinical studies in hormonal therapy for high and low-grade ovarian cancer have shown favorable response in patients (71, 72), suggesting prospective clinical trials using the combination therapy with CDK4/6 inhibitors in these patient populations. Ribociclib is currently under Phase II trial (NCT03008408) for use in combination with letrozole and the mTOR inhibitor temsirolimus in patients with advanced and recurrent endometrial cancer (73). Ribociclib is also under Phase II trial (NCT02657928) for use in combination with letrozole to treat patients with estrogen receptor-positive ovarian fallopian tube, primary peritoneal, or endometrial cancer. Furthermore, ribociclib in combination with platinum-based chemotherapy is currently under Phase I trial for patients with recurrent platinum-sensitive ovarian cancer (NCT03056833).

Abrogation of CDK1/2 activity by small molecule inhibitors mainly leads to impediment of S and G2/M phase progression. Several small molecule inhibitors of CDK1/2 have been evaluated in early and late stages of clinical trials. None of these inhibitors have been approved by FDA thus far. CDK1/2 activity is required for DSB end resection and HR repair (34–37). Given the importance of HR repair in ovarian cancer and, to a lesser extent, other gynecologic cancers, drug combination strategies to exploit CDK1/2 deserve further investigation. Cumulative preclinical data have demonstrated that CDK inhibition augments the anticancer efficacy of DNA damaging modalities through impairment of HR repair. It has been shown that depletion of CDK1 or pharmacological inhibition of CDK1 sensitizes BRCA-proficient breast cancer to a PARP inhibitor (74, 75). Roscovitine (Seliciclib) is one of the first CDK1/2 inhibitors identified (76) and widely demonstrated to sensitize various cancers to DNA damaging agents and radiation (77, 78). The chemo- and radio-sensitizing effects of roscovitine are consistent with its ability to block DSB end resection and impair HR repair (35, 37, 79). Roscovitine is currently under Phase I clinical trial in combination with the nucleoside analog sapacitabine in patients with advanced solid tumors including pancreatic, breast, and ovarian cancers (NCT00999401) (80). The side effects of roscovitine have been reported to be neutropenia, elevated transaminase and bilirubin, hyperglycemia, and abdominal pain.

Currently available small molecule inhibitors of CDK1/2 exhibit specificity toward CDK1, CDK2, or both, and, to relatively minor extent, other CDKs. Thus far, most CDK1/2-specific inhibitors are still under preclinical investigation or at early stages of clinical trials. Dinaciclib (SCH727965) is a dual CDK1 and CDK2 inhibitor and has been demonstrated to inhibit HR repair and sensitize multiple myeloma cells to the PARP inhibitor veliparib (81, 82). Clinical studies of dinaciclib as monotherapy revealed adverse events including hypotension, diarrhea, nausea, vomiting, and fatigue (83, 84). Ro-3306 is CDK1-specific inhibitor that arrests cell cycle at the G2 to M transition and induces apoptosis in cancer cells with prolonged exposure (85). It has been shown to impair HR repair and sensitize BRCA-proficient breast cancer to PARP inhibitors (74, 75). BMS-387032 (SNS-032), a CDK2-specific inhibitor, has been identified by high-throughput screening and demonstrated to have broad spectrum anti-proliferative activity against a panel of cancer cell lines (86). It has also been shown to synergize with cytarabine (ara-C), a DNA damaging antimetabolite drug, to treat AML cells (87). PHA-793887 is a CDK2-specific inhibitor that exhibits favorable efficacy against cancer xenografts and disrupts DSB end resection after radiation (38, 88). AZD5438, a dual CDK1 and CDK2 inhibitor, enhances radiosensitivity of non-small cell lung cancer by impairing HR repair of DSBs (89, 90). CDK12, a member of CDK subfamilies involved in transcriptional regulation but not cell cycle progression (91), has been implicated in contribution to HR repair gene expression and PARP inhibition resistance in high grade serous ovarian cancer (92). In addition to its ability to inhibit CDK1 and CDK2, dinaciclib abrogates CDK12 activity and suppresses the expression of BRCA1, BRCA2, and Rad5, thereby sensitizing BRCA-wild type triple negative breast cancer to PARP inhibition (93). Since the majority of CDK1/2 inhibitors equally target CDK1 and CDK2 as well as other CDKs, emphasis should be made on the development and comparative testing of CDK1-specific or CDK2-specific inhibitors, which would potentially minimize toxicity associated with cell cycle arrest and transcriptional suppression in normal cells. In addition, optimizing the dosing schedule in patients to mitigate side effects would facilitate the clinical development of CDK1/2 inhibitors moving forward.

## Cdc25 inhibitors

The activity of Cdc25 phosphatases is essential to remove the inhibitory phosphorylation of CDK1/2 and thus promote S and G2 phase progression (Figure 1). Therefore, targeted ablation of Cdc25s has been reportedly effective against a variety of cancer types, including ovarian and endometrial cancers (94). Thus, it is of great interest to develop small molecule inhibitors of Cdc25s for cancer therapies. Many quinonoid-based Cdc25 inhibitors have been identified and demonstrated to inhibit proliferation of cancer cells in a manner similar to CDK1/2 inhibitors. However, clinical development of quinonoid-based Cdc25 inhibitors has encountered major hurdles because the mechanism of action involves ROS generation and covalent modification of Cdc25s (95). LGH00031 is an irreversible quinonoid Cdc25 inhibitor that inhibits proliferation and causes G2 arrest of several cancer cell lines by increasing phosphorylation of CDK1 at Tyr15 (96). BN82002 is a non-quinonoid inhibitor of Cdc25 phosphatases that causes accumulation of inhibitory phosphorylation of CDK1, impediment of cell cycle progression, and inhibition of tumor growth *in vitro* and *in vivo* (97). LB100, a non-quinonoid cdc25 inhibitor, has been demonstrated to impair HR repair and sensitize ovarian and other cancer cells to radiation, daunorubicin, and cisplatin (98–102). Clinical side effects have yet been studied for this class of inhibitors. The prospect of this class of small molecule compounds lies in the successful development of non-covalent and bioavailable Cdc25 inhibitors. Cdc25s are known to overexpress at a high rate in many cancers including breast, ovarian, and endometrial cancers (103). A recent study has revealed that Cdc25 inhibitors effectively target the triple negative breast cancer refractory to CDK4/6 and CDK2 inhibition (104). Given the importance of Cdc25s in cell cycle progression and oncogenic properties, preclinical and clinical development of Cdc25 inhibitors promises invigorating advances in future gynecologic cancer therapy.

## RNR inhibitors

Triapine (3-aminopyridine-2-carboxaldehyde thiosemicar-bazone) is a potent small molecule inhibitor of ribonucleotide reductase (RNR) (105, 106). Our laboratory has identified that triapine indirectly causes CDK inhibition which leads to impairment of HR repair (28, 107). Triapine is 1,000 times more potent than hydroxyurea (105, 108). Hydroxyurea is a FDA-approved RNR inhibitor for use in combination with radiation to treat patients with locally advanced head and neck cancers (109). RNR is a heteromeric enzyme consisting of R2 and R1 subunits during the S phase of the cell cycle, and of p53R2 and R1 subunits upon DNA damage (110, 111). Enzymatically active catalyzes the rate-limiting step in the conversion of ribonucleoside diphosphates (NDPs) into corresponding deoxyribonucleoside diphosphates (dNDPs), the immediate precursors of deoxyribonucleoside triphosphates (dNTPs) essential for replication and repair (112). Triapine quenches the tyrosyl radical in the R2/p53R2 subunit of RNR, thereby leading to enzymatic inactivation (113–115). Therefore, treatment of cells with triapine promptly causes depletion of purine nucleotides/dNTPs and stalls replicative synthesis (32, 116). Prolonged exposure to triapine causes cumulative collapsed replication forks and DSBs that lead to activation of apoptotic pathways (117).

Triapine is known to hinder S phase progression and interfere with DNA repair processes primarily due to depletion of dNTPs for DNA synthesis. We have previously demonstrated that triapine impairs HR repair of DSBs and sensitizes BRCA-wild type epithelial ovarian cancer (EOC) to PARP inhibitor, platinum drugs, and topoisomerase II inhibitors (28, 107). Our mechanistic studies elucidate that triapine causes activation of Chk1 which in turn blocks CDK-mediated phosphorylation of CtIP. Because phosphorylated CtIP is required to stimulate the endonuclease activity of the MRN complex, DSB end resection is abrogated, leading to impairment of HR. siRNA-mediated silencing of R2 subunit of RNR in EOC cells corroborates the inhibitory effects of triapine on HR (28, 107). Furthermore, research from Ramsden's group independently reports that stimulation of RNR activity promotes HR and suppression of RNR activity by hydroxyurea inhibits HR (118). Nevertheless, our findings provide potential explanations for preclinical and clinical observations that triapine effectively sensitizes cancer cells to radiation and DNA damaging modalities (28, 107, 108, 119, 120).

In clinical trials triapine exhibits only moderate anticancer activity as a single agent. With more than 90% clinical response rates in phase I/II studies (119, 121, 122), triapine in combination with cisplatin and radiation therapy is currently under multi-center, randomized phase II clinical trials for treatment of advanced cervical and vaginal cancers (NCT02466971). Clinically, triapine is very tolerable to patients. Due to its strong iron-chelation property, triapine causes notable side effect of methemoglobinemia and dyspnea (123). Other adverse events include nausea, diarrhea, anemia, leukopenia, and thrombocytopenia (124). However, these side effects are generally manageable as the plasma half-life of triapine is short (125) and the methemoglobinemia antidote, methylene blue, is available (123). Nevertheless, abrogation of RNR activity by triapine mirrors inhibition of CDKs and impairment of HR repair. The rationale has immense potential to devise pragmatic combination strategies with other DNA damaging modalities, such as PARP inhibitors, to improve the patient outcomes of gynecologic cancer therapy.

## Concluding remarks

Defects in cell cycle regulation represent the vulnerability of cancers which offers an excellent opportunity for therapeutic intervention. The successful implementation of combined CDK4/6 inhibitors and hormonal/endocrine therapy in clinical practice for breast cancer lays the groundwork for other CDK and CDK-associated inhibitors currently under development. Like the lessons learned from conventional chemotherapy, targeting inhibition of CDK with small molecule inhibitors alone often falls short of the promise. Besides a better understanding of hormone-mediated cell cycle progression, advent of molecular insights into the connection between CDK activity and DNA repair provides additional rationale for designs of combination therapies for cancers. Gynecologic cancers, especially ovarian cancer, exhibit hypersensitivity to DNA damaging modalities including platinum drugs and PARP inhibitors when the HR repair capacity is compromised (126). Given that cancers rely on active CDK to perpetuate cell proliferation at all costs, abrogation of CDK-driven HR repair create synthetic lethality to evoke apoptosis and allow effective elimination of cancers with DNA damaging agents. However, implementation of such small molecule inhibitors of CDKs is just in the dawn of targeted therapy for gynecologic cancers. In conclusion, continuing efforts to discover CDK or CDK-associated inhibitors and their synthetic lethal combinations with novel DNA damaging modalities, such as PARP inhibitors, will provide tremendous advance in therapeutic approaches and hold promise in successful treatment of gynecologic cancers.

## Author contributions

All authors listed have made a substantial, direct and intellectual contribution to the work, and approved it for publication.

### Conflict of interest statement

The authors declare that the research was conducted in the absence of any commercial or financial relationships that could be construed as a potential conflict of interest.
